# Integrative Gene Regulatory Network Analysis Reveals Light-Induced Regional Gene Expression Phase Shift Programs in the Mouse Suprachiasmatic Nucleus

**DOI:** 10.1371/journal.pone.0037833

**Published:** 2012-05-25

**Authors:** Haisun Zhu, Rajanikanth Vadigepalli, Rachel Rafferty, Gregory E. Gonye, David R. Weaver, James S. Schwaber

**Affiliations:** 1 Daniel Baugh Institute, Thomas Jefferson University, Philadelphia, Pennsylvania, United States of America; 2 Department of Neurobiology, University of Massachusetts Medical School, Worcester, Massachusetts, United States of America; Niels Bohr Institute, Denmark

## Abstract

We use the multigenic pattern of gene expression across suprachiasmatic nuclei (SCN) regions and time to understand the dynamics within the SCN in response to a circadian phase-resetting light pulse. Global gene expression studies of the SCN indicate that circadian functions like phase resetting are complex multigenic processes. While the molecular dynamics of phase resetting are not well understood, it is clear they involve a “functional gene expression program”, e.g., the coordinated behavior of functionally related genes in space and time. In the present study we selected a set of 89 of these functionally related genes in order to further understand this multigenic program. By use of high-throughput qPCR we studied 52 small samples taken by anatomically precise laser capture from within the core and shell SCN regions, and taken at time points with and without phase resetting light exposure. The results show striking regional differences in light response to be present in the mouse SCN. By using network-based analyses, we are able to establish a highly specific multigenic correlation between genes expressed in response to light at night and genes normally activated during the day. The light pulse triggers a complex and highly coordinated network of gene regulation. The largest differences marking neuroanatomical location are in transmitter receptors, and the largest time-dependent differences occur in clock-related genes. Nighttime phase resetting appears to recruit transcriptional regulatory processes normally active in the day. This program, or mechanism, causes the pattern of core region gene expression to transiently shift to become more like that of the shell region.

## Introduction

Our goal is to understand the coordinated gene expression program by which light induces a phase shift in the circadian rhythm of the master clock (“clock resetting”) that resides in the suprachiasmatic nucleus (SCN). Over recent years we have learned that this system is particularly resilient to single gene manipulations. Rather than arising from single causal genes it is a multigenic function engaging multiple genes in variable gene networks. The behavior of some of these genes has been identified by genomic-scale gene expression studies [1], [2]. In the same time frame other studies have shown significant regional differences in light-response within the SCN, suggesting coupled neuronal network interactions between SCN sub-regions containing neurons with distinct patterns of response. These prior results indicate that clock function in phase shift emerges from complex interactions involving multigenic networks with regionally distinct gene expression programs. Exploring this hypothesis of regionally distinct expression programs requires (1) identification of a substantial panel of genes involved in the response network, (2) simultaneous, quantitatively precise, high-throughput measurement of their response pattern over time (3) within distinct SCN regions and (4) extensive computational analysis of the resulting high-dimensional datasets. These requirements motivate the present study in which we describe the organization of the integrated gene network response in phase shift within distinct SCN subregions.

The multigenic character of both the clock and the downstream genes affected by resetting is seen in unbiased global microarray studies (e.g. [1], [2], [3], [4], [5], [6], [7]). We have analyzed the results from these prior global studies to derive a phase-resetting relevant panel of 89 genes useful for our study of their integrated network behavior as a gene expression program for phase-shifting to light exposure at night. For example, light-induced phase shifting induces immediate early genes (IEGs) simultaneously [8], including *fos* and *jun*, as well as the clock genes *per1* and *per2* [9], [10], [11]. These IEGs and the proteins they encode are thought to activate or influence gene expression cascades involving neuropeptides and membrane receptors, and possibly genes for signaling pathways.

We here aim to understand the entrainment of these genes by light in complex temporal gene networks, which is an important and difficult problem (e.g. [12], [13], [14], [15]). Further, we aim to begin the study of distinct regional responses. There may be several SCN regions with distinct responses [14], [16], [17], [18] but we initially focus on two, the shell and the core regions. Distinct responses in these regions have been well established most clearly in species such as the hamster and rat [19], [20], [21]. However, studies of single early immediate genes (i.e. *c-Fos*, *Per*) following light exposure in mouse are ambiguous as to regional localization [22], [23], [24]. Simultaneous measures of a panel of genes as in the present study may better disambiguate regional responses in the mouse. At the same time we intend to sample very conservative, restricted regions based on anatomical markers within the larger mouse core and shell regions in order to be certain to divide these two subnuclei of mouse SCN.

Data are derived from RNA samples of both the core and shell regions from 18 animals, allowing us to use qPCR to monitor the expression of 89 genes (and 3 control genes) in a functional program from each biological sample, region, time, and in response to acute light exposure at night. These data lend analytic power to the description of circadian gene expression and its orchestrated regulation as a program. We used two time points: one at night, known to be sensitive to light-induced phase resetting, and one during the day, when light exposure is ineffective. In this, we expect to observe previously reported rhythmic profiles for several genes, and observe how temporal changes in their expression relate to dynamic expression changes in other functionally related genes. The results systematically describe the time- and region-specific regulation of the expression program in response to a phase-shifting light exposure. We analyzed the orchestration of the circadian gene expression program in response to a phase-shifting light exposure at night using computational and mathematical descriptions. Principal component analysis (PCA) highlights differences that extend across core-shell regions, times and treatments. We also have used our Promoter Analysis Interaction Network Tool (PAINT) to predict gene regulatory networks involved in producing observed circadian expression changes. Functional annotation analysis was used to evaluate patterns in temporal relationships. The results reveal a complex and anatomically specific phase resetting program within SCN, with similarities and with important differences to spontaneous daily changes in gene expression within the SCN, and suggest that nighttime phase resetting recruits transcriptional regulatory processes normally active in the day.

## Results

### Gene Selection and Experimental Design

**Figure 1:**
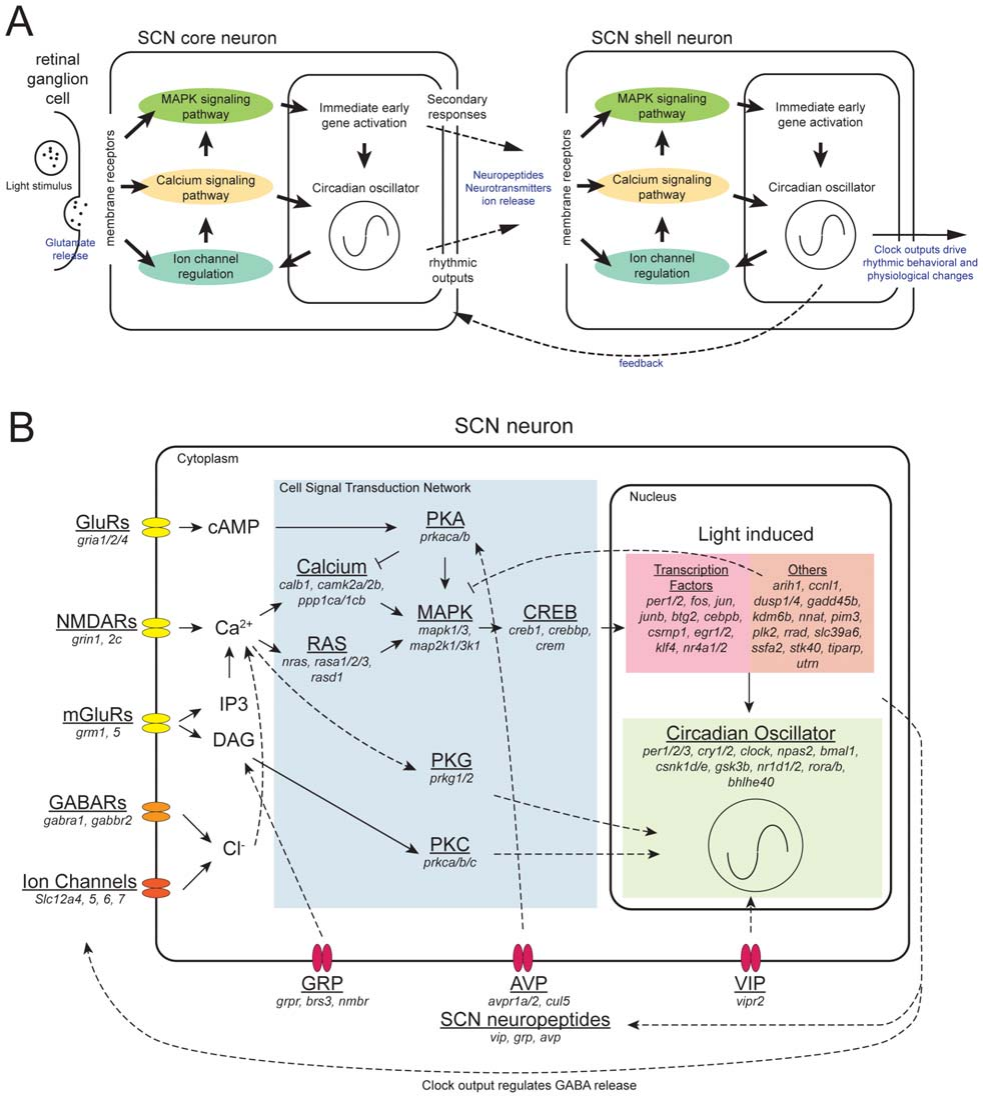
Light pathway within the SCN and genes that involved in the process. A) An illustration of light signal's progression through the core and shell of the SCN. Major functional groups are highlighted including membrane receptors, cellular signal pathways, immediate early gene activations, and circadian oscillator. B) A consolidated view of an SCN neuron. Genes listed were used in the qPCR assay, and focus on the circadian clock and genes involved in mediating its response to light. Definitive gene functions are shown in solid arrows. Implied gene functions are shown in dashed arrows.

Our goals are to understand the dynamic changes in the expression program of a system of genes within the SCN in response to a light pulse, and to provide a systematic network analysis of SCN cellular oscillators. In order to assess the state of the clock as well as the effect of light on the SCN, we selected 89 genes to best represent key pathways involved in circadian photoreception (Figure 1B) for quantitative expression profiling together with 3 housekeeping genes (*gapdh*, *tbp*, and *actb*; Table S1). These genes include the neuropeptides, arginine vasopressin (*avp*), gastrin releasing peptide (*grp*), and vasoactive intestinal peptide (*vip*), which allow us to distinguish SCN from surrounding non-SCN tissue, as well as to serve as marker genes confirming our sub-regional collection of the SCN (Figure S1). We have also included an extensive set of genes that are directly responsible for generating the molecular oscillation within individual SCN neurons [25], [26]. Light-induced expression of *per1* and *per2* appears to play an important role in photic resetting [10], [11], [23], so these were included in the assay set to demonstrate that the light stimulus had been perceived within the SCN. Previous studies have identified several immediate early genes (IEGs), including *fos* and *jun* that are rapidly induced by light [27], [28], [29], [30]. We included these genes to monitor the light response inside the SCN. We have also included glutamate and neuropeptide receptors, to assess potential neuromodulation within SCN neurons. In addition, our list includes many genes involved in intracellular signal transduction; changes in levels of expression of these genes in response to a light pulse would suggest potential use of these signaling pathways, and possible mechanisms of phase shifting in the SCN. Overall, our gene list is rich in transcription factors, neuropeptides, kinases, phosphatases, and G-protein coupled receptors. This combination gives us a dynamic overview of molecular responses within mouse SCN. (Table S1 summarizes the literature evidence for rhythmicity and/or induction of expression by light-at-night for each gene).

**Figure 2:**
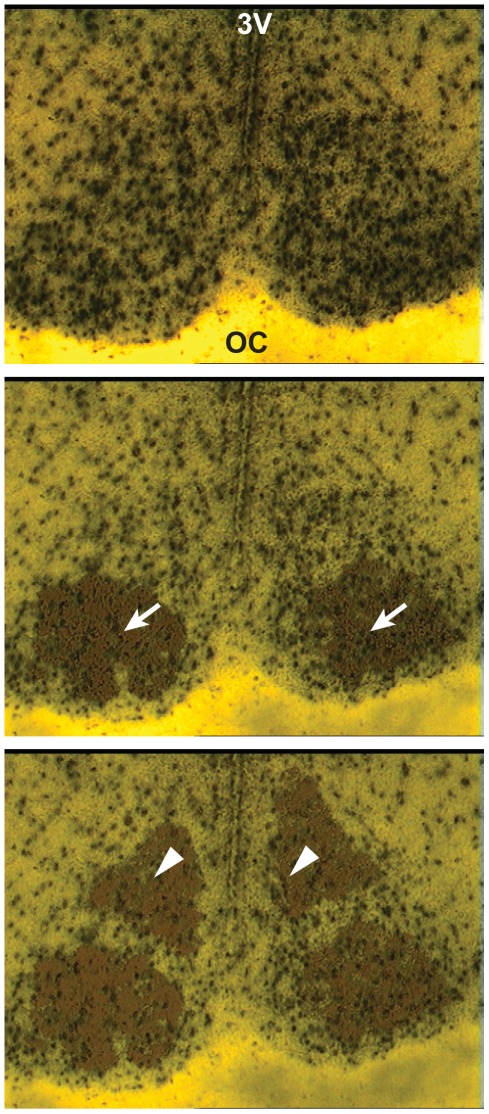
Laser Capture Microdissection in Mouse SCN. A cryostat section through the mouse SCN was stained with hematoxylin and counterstained with eosin. Top, dry-mounted brain section before LCM capture. The SCN is identified by the location of the optic chiasm (OC) and the third ventricle (3v) as well as higher neuronal density apparent by staining. Middle, the core region (arrows, ventral-lateral part of the SCN) has been captured. Bottom, the shell region (arrowheads; dorsal-medial part of the SCN has been captured.

In order to track anatomic differences in gene expression, we collected samples from core and shell of the SCN, and adjacent hypothalamis using laser capture microdissection (LCM) (Figure 2) based on existing literatures [17], [18], [22], [31]. The validity of these regional collections was confirmed by statistical significant high levels of the neuropeptide markers genes: *vip*, *grp*, in the core and shell-specific *avp* expression (Figure S1). A one-hour light pulse (LP) was given at ZT 14 (Zeitgeber time, 2 hours into the dark period) to induce maximum phase delay [4]; hypothalamic samples were extracted at the end of the light pulse (ZT 15+LP). Samples were also collected from control (non-light pulsed) animals (ZT 15), as well as during the day (ZT 6). These groups allow us to identify multiple variables of interest: first, specific responses to light pulse (ZT 15 vs. ZT 15+LP); second, rhythmic regulation (ZT 6 vs. ZT 15); and third, similarity and differences between the two conditions under light (ZT 6 vs. ZT 15+LP).

### Distinct expression profiles of the SCN

**Figure 3:**
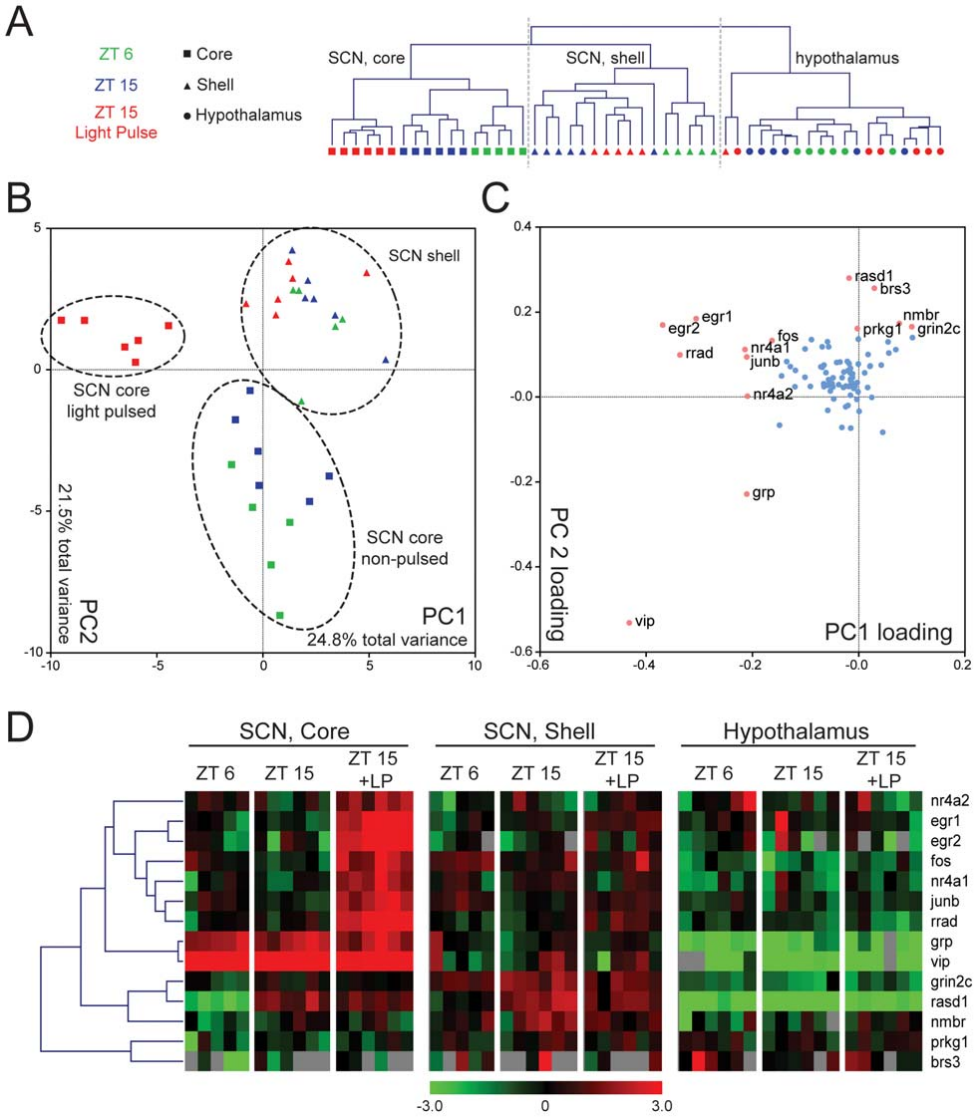
Hierarchical clustering of laser captured mouse SCN samples and principal component analysis. Results from qPCR were normalized by calculating −ΔC_T_ using the average C_T_ of *gapdh*, *tbp*, and *actb* as control. A) Unsupervised clustering of samples based on similar expression profiles showing separation between brain regions, times, and light treatment. Gray bars represent failed PCR reactions. B) PCA of the SCN samples in PC1 and PC2 space. Colors and shapes represent the same as in A). Clear separations between the SCN core and shell samples as well as the light pulse core samples are circled. C) Calculated eigenvector values of the first two 2 PCs. Each dot represents a gene. Genes with greater influence on PCA (loading factor greater than 0.15 or less than −0.15) are shown in pink with the names listed. D) Expression of these genes with more extreme loading factors are also shown in hierarchical cluster based on Pearson Correlation. Each row represents a gene and each column represents one LCM sample. Samples are grouped by regions (core and shell of the SCN, hypothalamus), then by time of collection (ZT 6, ZT 15, and ZT 15 with light pulse (ZT 15+LP)). Color bar represent a median centered −ΔC_T_ range of −3 to 3.

We first compared gene expression in all samples for the selected gene set. These results are hierarchically clustered (Figure 3A). The neuroanatomical regions where our samples were collected contribute the most to the differences in our data; the hypothalamus samples separate from the SCN samples, with the exception of one shell sample. The core and shell of the SCN further separate from each other. Within the SCN core region, the expression patterns of the three time point groups (ZT 6, ZT 15, and ZT 15+LP) are also distinct, with the light pulsed samples showing the most differences compared to the other two groups. The shell samples also display temporal variations; however, the difference between ZT 15 and ZT 15+LP is less prominent. Hypothalamic samples, on the other hand, do not display any significant difference among the time points.

To ensure that the choice of control genes did not affect the outcome, we also tested normalization using each of the three control genes individually. The resulting sample cluster maps were similar (Figure S2).

To further investigate differential expression between the three conditions within the SCN, we used principal component analysis (PCA). PCA enables visual representation of complex quantitative data sets with many interacting variables that highlight patterns of similarity and differences between groups. The main aspect of PCA is data reduction, that here is used to identify specific genes that contribute most to the observed differences between samples. PCA quantitatively evaluates the level of gene expression of the 89 assays in each sample, and plots each on newly defined axes that are most capable of describing the variability among samples. The first axis, called principle component 1 (PC1), is set to lie along the direction of most variation, and the original data are rotated so that they are plotted along this axis. The second component, PC2, is oriented orthogonally to PC1 and captures most of the remaining variation. As a result, the assigned position of the sample in the PCA plot represents the sum total of gene expression for the sample. When samples in the PCA are grouped together according to experimental condition, it can be said that the gene expression within samples exposed to those conditions are more similar to each other than they are to other experimental conditions. It is also possible to identify the genes that contribute most to differences between experimental conditions or groups by referring to the corresponding loading coefficients of each PC axis. These coefficients (i.e., weights of each gene that make up that PC) quantify the degree to which a particular gene from the original data set contributes to differences between samples. Consequently, when samples group together according to experimental condition, the genes with highest contribution to the PC have the largest coefficients (i.e., weights) and most significantly contribute to the observed separation.

In the present study, PC1 comprised 24.8% of observed variance in gene expression, and specifically differentiated the light-pulsed core samples from the remaining SCN samples (Figure 3B). Examination of the loading coefficients of the genes that contributed to the differentiation of the light-pulsed core samples indicated that the IEGs (*fos*, *junb*, *egr1*, *egr2*, *nr4a1*, *nr4a2*, and *rrad*) had substantial influence on this sample group separation along PC1 (Figure 3C). This can also be appreciated in the hierarchical clustering of the genes (Figure 3D). PC2 accounted for 21.5% of total variance and loosely separates SCN core samples from shell samples. Of note, the light-pulsed core samples clustered with the shell samples along the PC2 axis (Figure 3B), suggesting that light-pulsed core is more “shell-like” following light exposure at night.

**Figure 4:**
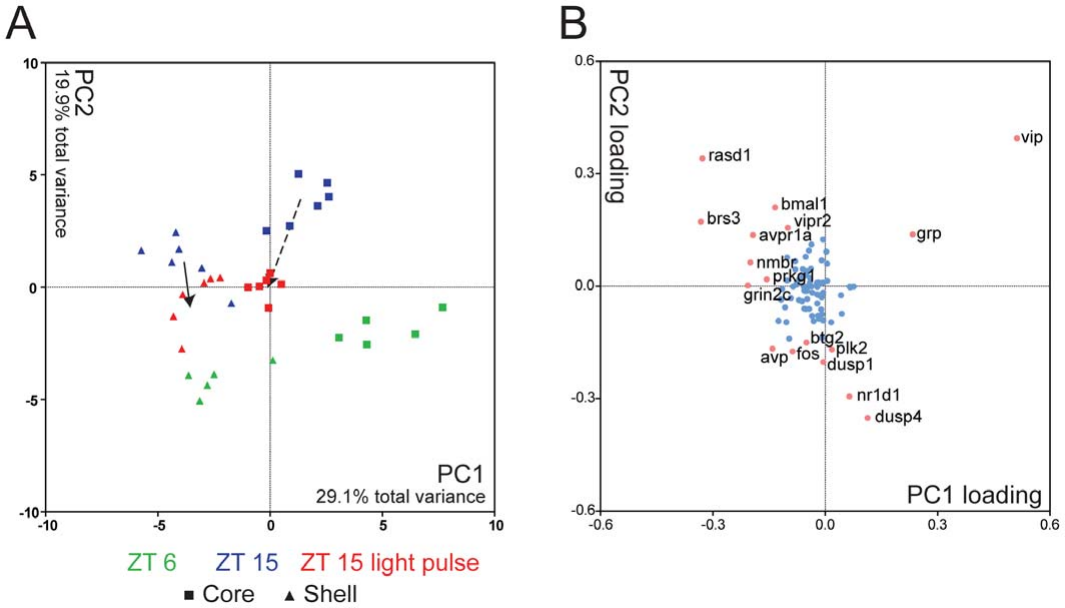
Distinct temporal and spatial expression profiles in core and shell of the SCN and effects of light pulse. A) PCA of non-light-pulsed SCN core and shell samples is shown in PC1 and PC2 space. Clear separations of core and shell samples by PC1 values as well as day (ZT 6) and night (ZT 15) samples by PC2 values can be seen. Light pulsed samples (red, ZT 15+LP) are projected based on calculated eigenvectors of the first 2 PCs. Solid arrow indicates trend of light-pulsed shell samples. Dashed arrow indicates trend of light-pulsed core samples. B) Calculated eigenvector values of first two 2 PCs. Each dot represents a gene. Genes with greater influence on PCA (loading factor greater than 0.15 or less than −0.15) are shown in pink with the names listed.

Due to the strong effect of the light pulse, subtler differences in day vs. night gene expression were masked when using all SCN samples in the PCA. However, the temporal expression patterns can be highlighted by performing a second PCA with only on ZT 6 and ZT 15 (in dark) SCN samples, as shown in Figure 4. In this case, PC1 (29.1% of total variance) separates core from shell samples (Figure 4A), while PC2 (19.9% of total variance) separates day (ZT 6) and night (ZT 15) samples. Genes with a loading coefficient more extreme than +/−0.15 contribute significantly to the separation of samples. We found that SCN neuropeptides (*vip*, *grp*, and *avp*) and their receptors (*brs3* and *avpr1a*) contributed strongly to neuroanatomic variation (PC1, Figure 4B). In addition, we identified differential expression of *grin2c*, a glutamate receptor subunit, and *prkg1*, cGMP dependent protein kinase, which contributed exclusively to PC1. This observation suggests that NMDA receptor and protein kinase G related signaling pathways are also a contributing component to the unique molecular phenotype distinguishing core and shell. In contrast, circadian clock components (*bmal1* and *nr1d1*) and clock regulated genes (*fos*, *vip*, *avp*, *rasd1*, *dusp1*, and *dusp4*) primarily affected PC2, accounting for temporal differences in sample expression (Figure 4B; see also Figure S3 and Table S2). Similarly, temporal differences in gene expression were seen in putative immediate early genes *plk2* and *btg2* (PC2).

In order to verify our earlier observation (Figure 3B) that the light pulse makes the expression program in the night samples resemble that in day samples, we projected the light-pulsed core and shell samples onto the PCA plot derived from ZT 6 and ZT 15 samples. In the shell samples, the light pulse caused the expression profile to move from the original position (ZT 15 without a light pulse) downward along PC2, toward day, making the ZT 15+LP samples appear transitional (Figure 4A, solid arrow). In contrast, the light-pulsed core samples not only showed movement vertically (downward) along PC2, from ZT 15 toward ZT 6, but they also moved leftward along PC1 toward a more shell-like expression pattern (Figure 4A, dashed arrow).

### Temporal and regional patterns of differential expression in the SCN

**Figure 5:**
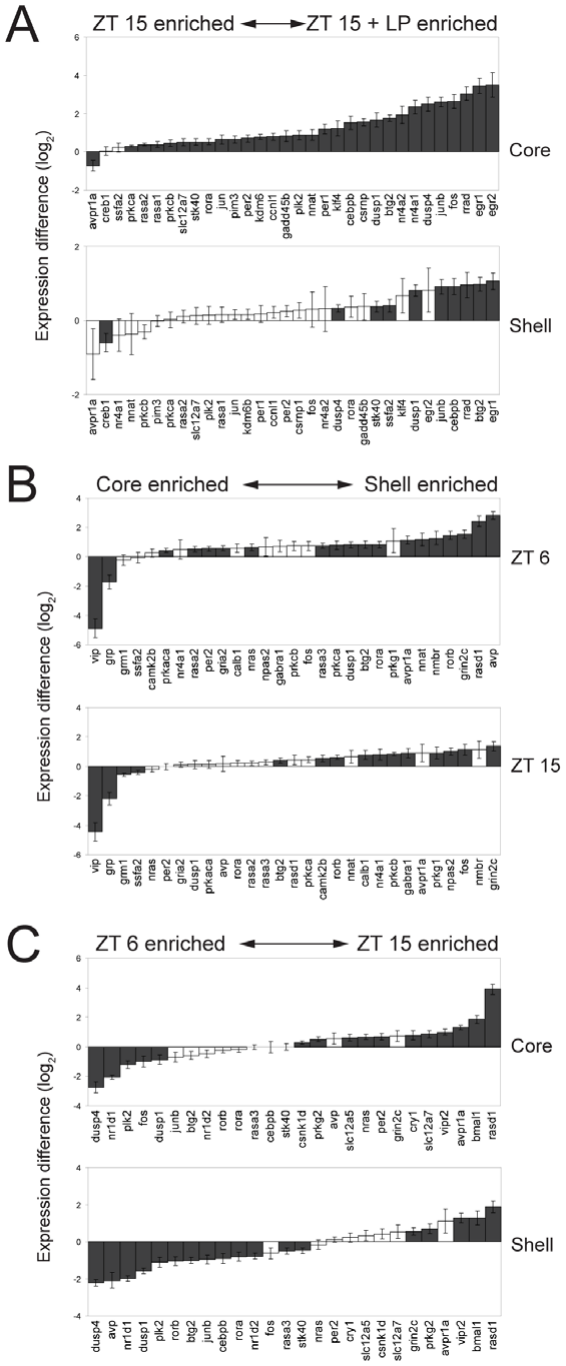
Differential Expression in SCN. Expression differences (−ΔΔC_T_) are shown. A) The presence or absence of a light pulse at ZT 15 significantly affected the expression of 32 genes in the SCN. The genes are ordered based on the −ΔΔC_T_ values. Genes significantly expressed in either sub-panel are shown in both sub-panels. B) A total of 29 genes showed significant differential expression between core and shell of the SCN. C) A total of 26 genes showed significant differences between day (ZT 6) and night (ZT 15). Black columns indicate significant differences (ANOVA with post hoc *t*-test, p<0.05). Error bars indicate standard errors.

The PCA provides an estimate of the behavior of the samples and their gene expression programs as a whole. More specific information about gene expression changes can be obtained by directly comparing gene expression levels between samples. We first compared the gene expression in the core and shell regions of the SCN following exposure to a 1-hour light pulse at night (Figure 5A). Of the 32 genes that showed significant changes (ANOVA then followed up with individual comparisons using post hoc *t*-test p<0.05) in expression levels between dark-housed controls (ZT 15) and light pulsed mice (ZT 15+LP), 30 were increased by light (Figure 5A). These include 21 genes that were induced only in the core, 8 genes that were elevated in both core and shell, and one gene (*ssfa2*) induced exclusively in shell. (Statistical results for all 89 genes are shown in Figure S3A; values are in Table S2). The prevalence of gene induction in core by light may reflect the neuroanatomic connections, and the core's role as the direct receiver of input from the retina which relays integrated and filtered information on to the shell [16], [32], [33].

The majority of the previously identified light-induced IEGs (Table S1) were induced in the core of the SCN following light pulse. In addition, we also observed light-sensitivity in *rasa1*, *rasa2*, *rora*, *pakca*, *parkcb*, and *slc12a7*, genes not previously known to be induced by light. Two genes appeared to be repressed by light: *creb1* and *avpr1a*. *Creb1* is down regulated in the shell only; *Avpr1a* is down regulated in both core and shell (although only core repression was significant).

PCA (Figure 4A) also separates the samples anatomically, with a distinct difference in gene expression between the core and shell samples. Comparing core and shell samples on a gene-by-gene basis, we find 29 genes with differential expression between these two regions (ANOVA with post hoc *t*-tests, *p*<0.05, Figure 5B, Figure S3B). Most of these genes are expressed at higher levels in the shell than in the core (shell-enriched); in fact, only 4 genes show higher expression in core than shell. Of those 4, *vip* and *grp* showed significantly higher expression levels in the core at both time points, as expected; these genes encode SCN neuropeptides selected for inclusion because of their known core localization. The other two core-enriched genes, *grm1* and *ssfa2*, were elevated slightly at ZT 15.

Of the 25 shell-enriched genes, three (*grin2c*, *rorb*, and *btg2*) are more highly expressed in shell at both time points. *Avp*, a canonical shell marker, is more highly expressed only at ZT 6, consistent with this transcript's well-established rhythmic expression [34]. Additionally, 13 other genes (*avpr1a*, *dusp1*, *gria2*, *nnat*, *nmbr*, *nras*, *per2*, *prkaca*, *prkca*, *rasa2*, *rasa3*, *rasd1*, and *rora*) are more highly expressed in the shell than core at ZT 6. The remaining eight (*calb1*, *camk2b*, *fos*, *gabra1*, *npas2*, *nr4a1*, *prkcb*, and *prkg1*) show higher expression levels in the shell only at ZT 15.

Finally, we identified genes that showed temporal differences in their expression between ZT 6 and ZT 15 within each SCN core or shell region (Figure 5C, Figure S3C). Of the 26 genes that differed in expression level in either core or shell (ANOVA with post-hoc *t-*test, *p*<0.05), 14 had higher expression levels at ZT 6, while 12 genes were lower at ZT 6 than at ZT 15. Among the 14 genes that were higher at ZT 6, 4 genes (*dusp1*, *dusp4*, *nr1d1*, and *plk2*) were elevated in both SCN regions. One gene, *fos*, was higher at ZT 6 only in the core; the 9 remaining genes (*avp*, *rorb*, *btg2*, *junb*, *cebpb*, *rora*, *nr1d2*, *rasa3*, and *stk40*) were higher at ZT 6 in the shell. Of the 12 genes that show higher expression at ZT 15, 4 genes (*bmal1*, *rasd1*, *vipr2*, and *avpr1a*) are higher at ZT 15 than at ZT 6 in both core and shell. 7 more genes (*cry1*, *csnk1d*, *nras*, *per2*, *prkg2*, *slc12a5*, *slc12a7*) were expressed at higher levels at ZT 15 only in the core. *Grin2c* is the lone gene of the twelve that showed higher levels of expression at ZT 15 in a shell-specific manner.

In summary, the differences in gene expression levels observed in the six possible combinations of treatment conditions (core vs. shell, ZT 6 vs. ZT 15, and ZT 15 vs. ZT 15+LP; Figure 5) are not uniformly distributed across these groups. When gene expression level is analyzed by these factors, two groups are remarkably underrepresented (as shown in Figure 5): there are only two genes that are expressed more highly at ZT 15, relative to ZT 15+LP, and there are only two to four genes that are enriched in core, relative to shell at either ZT 6 or ZT 15, and these genes overlap. Stated another way, the gene expression programs assessed by our analysis reveal higher levels of expression of many more genes in shell >core, and independently reveal many more genes are elevated at ZT 15+LP>ZT 15 in darkness. Only Panel 5C appears more evenly balanced, with similar numbers of genes enriched at each time (ZT 6 and ZT 15) in each of the two regions. The result of this analysis is four distinct, functionally defined gene sets: (1) shell-enriched genes, (2) genes elevated during the day, (3) genes elevated during the night, and (4) light-induced genes. Subsequent analysis sought to identify mechanisms for coordinated regulation within each of these gene sets.

### Functional correlation between diurnal regulation and light response during night in differentially expressed genes

**Figure 6:**
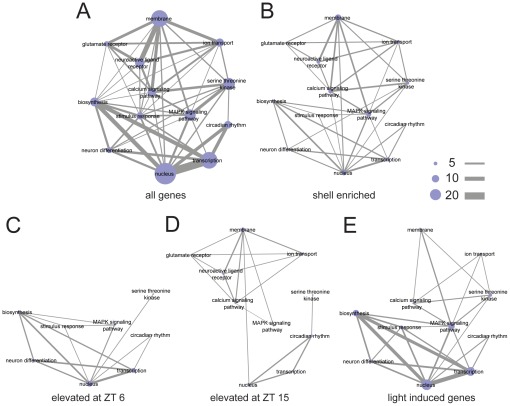
Functional networks of enriched gene sets in the SCN. The genes selected for qPCR were annotated using DAVID. Relevant functional terms were manually selected. A) Network representation of term interactions was generated using Cytoscape. The node size represents the number of genes that have been annotated to a particular term. The connection between the nodes means at least one gene is shared between the two terms with greater number of shared genes represented by thicker connections as the scale bars indicated. B) A network based on shell-enriched genes. C) A network represents the genes elevated during the day (ZT 6). D) A network represents the genes elevated during the night (ZT 15). E) A network based on light-induced gene only.

In order to understand the impact of light treatment, SCN region, and time on gene expression patterns within the SCN in terms of their functional relevance, here we present sets of differentially expressed genes as a network of functionally relevant annotation terms assigned using the Database for Annotation, Visualization and Integrated Discovery (DAVID; [35], [36]). The functional annotation network representing the entire set of 89 genes measured in this study is shown in Figure 6A; nodes represent the functional annotation terms, sized according to the number of genes associated with this term in our experiment. A connection between two terms indicates that at least one gene is assigned to both annotations, while the width of the connecting lines represent the number of shared genes. A detailed list of the DAVID annotated functional terms for each gene in our analysis are provided in Table S3. As illustrated in Figure 1, our gene set includes genes with a range of functions from membrane-bound receptors to nuclear transcription factors. Connecting these two groups are key signal transduction pathways, such as MAPK and pathways involved in calcium signaling, also represented in the dataset.

Using the complete network as a template, we mapped out the functional annotation network for four differentially expressed gene sets defined above (Figure 6B–E). Unrepresented functional groups (nodes) and node connections were removed. This allows us to emphasize related functional groupings of genes that are differentially expressed in a particular experimental condition.

Many genes among those analyzed are enriched in the SCN shell (Figure 5B). The functional annotation network of the 25 shell-enriched genes (Figure 6B) appears to be evenly distributed, as none of the selected functional terms are missing, and most node connections are preserved.

Unlike the shell-enriched network, the temporally regulated genes (Figure 6C, D) have distinct functional network representations. Genes elevated during the day (ZT 6, Figure 6C) are closely related with annotation terms for transcription, nucleus, as well as biosynthesis. In contrast, while genes elevated during the night (ZT 15, Figure 6D) also involve some regulators of transcription, the night gene set also emphasizes membrane-associated functional terms, such as glutamate receptors and ion transport that were not seen in the day-active genes. Elevated expression of membrane receptors and related genes at night might make the SCN more susceptible to outside stimuli such as the light-induced release of retinohypothalamic neurotransmitters during that time.

The light pulse-induced gene set forms a distinct functional network (Figure 6E), that in many ways resembles the day functional network (ZT 6; Figure 6C), but involves a larger number of genes associated with transcription, the MAPK signaling pathway, and biosynthesis. This is congruent with the PCA results, indicating that the light pulse is activating a day-like gene expression profile. The enriched functional association of light-induced genes with nucleus, biosynthesis and transcription fits well with the immediate early response following light exposure, feeding back to downstream pathways through transcription.

### Evidence of co-regulation of light-induced genes

To establish the roles of transcriptional regulation in light-induced activation, we performed transcriptional regulatory element (TRE) enrichment analysis on the promoters of light-induced genes using Promoter Analysis and Interaction Network Toolkit (PAINT, v.4.0) [37], [38]. Significantly enriched TRE, as determined by Fisher's Exact Test, indicates a role in co-regulation, and also suggests involvement of the corresponding transcription factors.

First, we examined the cAMP Response Element (CRE) and Serum Response Elements (SRE), as they are known to mediate immediate early gene activation through CRE binding protein (CREB) and Serum Response Factor (SRF), respectively. There are four types of CREs and three types of SREs in the promoters of the light-induced genes. However, none of the CRE sites show significant enrichment (Table 1), though three of the four are near significance (0.05<p<0.1). This is not surprising, as the statistical test is limited by our small but highly specific set of 89 genes, which is already heavily biased toward known light response genes. In fact, all four types of CRE sites are significantly enriched when comparing the promoters of all 89 genes to the entire mouse genome (Table S4) and the family of CRE sites as a whole is also significantly enriched in the light-induced genes when comparing to the 89 gene background (Table S5). Conversely, one (V$SRF_C) of the SRE sites did show significant enrichment (Table 1), but is only present in the promoters of 3 out of 30 light-induced genes. We have also identified several TREs belong to the AP1 family and the EGR family (Table 1). However, none of these TREs showed significant enrichment. For comparison, E-box like TREs, which are crucial for circadian regulation, do not appear to be enriched in the light induced genes set (Table 1).

**Table 1:** TRE enrichment analysis of light-induced genes.

TF	TRE	FET *p*-values*
CREB	V$CREB_02	0.140
	V$CREB_Q4_01	0.089
	V$TAXCREB_01	0.068
	V$TAXCREB_02	0.068
SRF	V$SRF_C	**0.036**
	V$SRF_Q4	0.262
	V$SRF_Q6	0.111
AP-1	V$AP1_Q2_01	0.563
	V$AP1_Q4_01	0.813
EGR	V$EGR1_01	0.109
	V$KROX_Q6	0.672
	V$CKROX_Q2	0.858
E-Box	V$ARNT_01	0.879
	V$EBOX_Q6_01	0.323
	V$MYCMAX_03	0.547
Others	V$E2F_03	**0.036**
	V$PAX3_B	**0.042**
	V$GABP_B	**0.011**

* Using TREs from the 89 genes tested as reference set.

**Figure 7:**
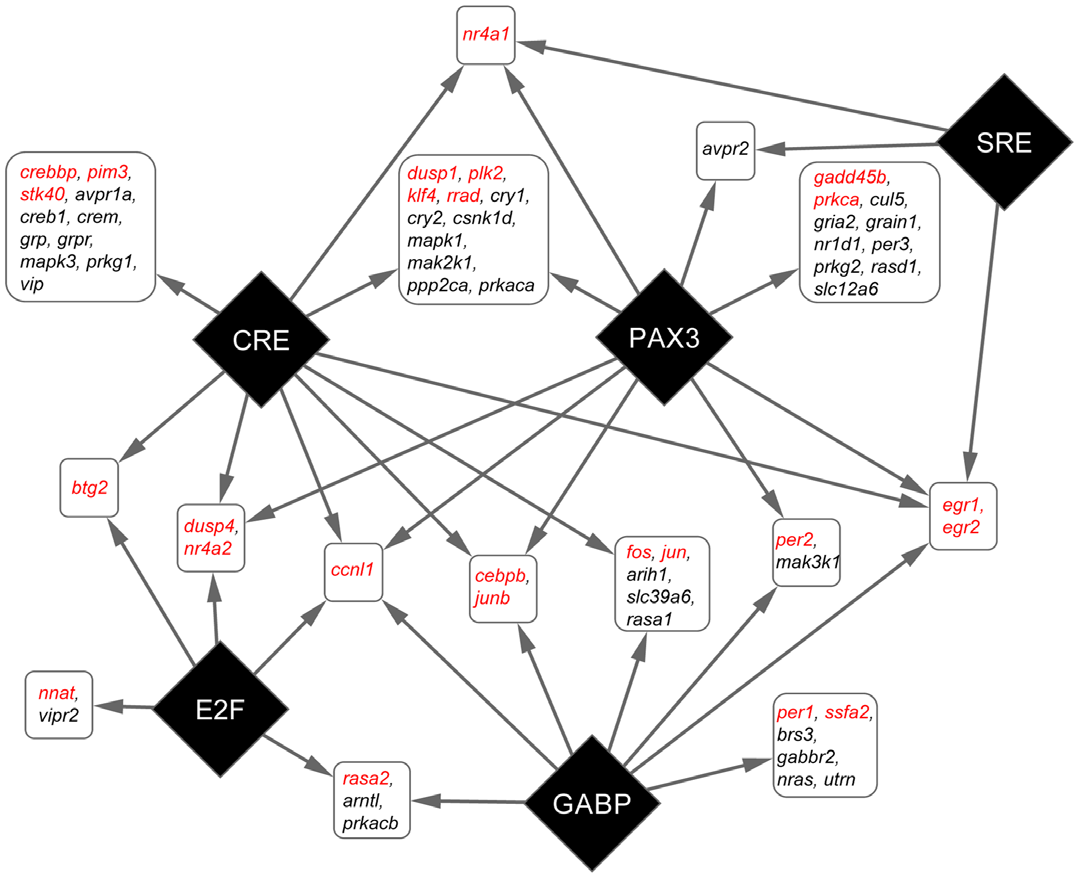
Co-regulation of light-induced gene activation. TRE families (diamonds) that are significantly enriched and genes (rounded rectangles) they regulate. Genes with promoters that shared the same TRE pattern are consolidated. Confirmed light induced genes are shown in red. Arrow lines indicate the presence of TRE in the promoter regions of a group of genes.

Besides the known families of TRE that involve light-induced activation, three TREs (V$E2F_03, V$PAX3_B, and V$GABP_B) are significantly enriched in promoters of light-induced genes (Table 1). These TREs, which are recognized by transcription factors E2F, paired box gene 3 (PAX3), and GA-binding protein (GABP), respectively, are known to regulate cell cycle, cell differentiation, and metabolism [39], [40], [41]. Their roles in light-induced activation are yet to be determined. We further examined the distribution of these significant enriched TREs in the promoters of light-induced genes. Our results show that while CRE sites are present in the promoters of many light-activated genes, having just CRE sites alone are not sufficient for light-activation. Many genes that have conserved CRE sites, such as *grp* and *vip*, do not respond to light exposure (Figure 7). GABP and PAX3 binding sites are often necessary in addition to the CRE sites (Figure 7). Many immediately early genes, such as *fos*, *jun*, *junb*, *egr1*, *egr2*, and *cebpb*, have both CRE and GABP binding sites. The latter four IEG's also carry PAX3 binding sites in their promoters. The presence of multiple enriched TRE sites suggests possible co-regulation, as well as synergistic activation, in addition to the known light-induction pathways.

## Discussion

Identifying SCN responses to light is an essential step toward understanding circadian clock neuronal integration and synchronization. Although many cellular and molecular functions of the clock have been identified, how the SCN functions as a whole, integrating environmental cues into a coordinated and rhythmic transcription response, is not fully understood. Here, we selected 89 genes as representative of an “SCN Phase Resetting Programs” and used this gene set to probe light response behavior of sub-regions of the SCN. We measured the expression of these genes in parallel, using microfluidic qPCR, which provide the most precision and sensitivity among available molecular tools [42]. We chose to examine SCN expression one hour after the onset of the light because we were not only interested in studying immediate early gene responses, but would also like to identify secondary gene activations within the SCN as well as differential responses between core and shell regions. The one hour time point for expression study proves to be highly informative (Figure 3, 5, 6) and also has been used widely in other light response studies of mammalian SCN [1], [6], [9], [10], [11], [43]. By examining the collective gene expression behaviors, we showed that transcriptional activities in the SCN are highly coordinated, as genes with similar functions are activated together at certain time of day or in response to the environmental perturbation of exposure to light at night. Our results show patterns of multigenic activity in response to light exposure that causes phase delay. This involves a transient movement of night expression patterns to resemble day expression patterns, and for core patterns to resemble shell patterns. We further showed that this kind of coordination in gene activity appears to be mediated through a gene regulatory network involving transcriptional co-regulation, using transcriptional element enrichment analysis.

The genes we selected for expression profiling represent a wide range of cellular functions that are related with circadian photoreception, ranging from membrane bound receptors to nuclear transcription factors (Figure 6A). Having such a functional network allows us to interpret our expression data in term of SCN neuronal behaviors. As such, we found many genes show diurnal variation in their expression levels and the peaks of the expression show high coordination based on the functions of the genes. Genes expressed at a higher level at ZT 6 are predominantly involved in transcription and biosynthesis (Figure 6C). In contrast, genes that show highe r levels at ZT 15 are rich in membrane receptors (Figure 6D), suggesting neurons at this time point might be more susceptible to extracellular stimuli, such as light exposure.

In addition to diurnal variations in gene functions, we also find a remarkable similarity in DAVID terms between the day-active genes and light-induced genes at night suggesting similar SCN neuronal behaviors (Figure 6C, E). However, despite significant overlap of individual transcripts (Figure 5, Table S6) and functional groupings, the actual gene regulatory functions are not likely to be the same under these different conditions; light-induced transcriptional activation, such as *fos* and *jun*, is both acute and high magnitude, while day-active expression profiles appear to be more moderate and prolonged. While it is possible that ambient light, present when our ZT 6 samples were collected might have increased the expression of “day-active genes” in the SCN, this seems unlikely. “Day-active” genes such as *per1* and *fos* are also rhythmically expressed in constant darkness with peak levels during subjective day at levels consistent with observed daytime levels in a regular light/dark cycle [29], [44], [45]. This suggests that regulation during the day is light independent. In addition, our results show that most ZT 6 elevation happens in the shell, which is not directly connected to retinal input. Therefore, it is possible that SCN neurons responding to light, utilized a rapid gene expression program to change their behavior to day-like, and thus achieved phase shift.

This possibility is supported by PCA analysis, as the light-pulsed ZT 15 samples mapped closer to the ZT 6 samples than ZT 15 samples (Figure 4). In addition, we found that the collective changes in gene expression make the light-pulsed core samples more “shell-like.” We also found other light-activated genes, such as *klf4* (also previously shown to be light induced in SCN [1], [2]), that show neither temporal variation nor SCN-specific enrichment in the absence of light input. The fact that these genes are induced suggests additional pathways are activated by light, and that these pathways may be involved in phase shifting.

In order to identify specific gene regulation in light-induced activation, we performed TRE enrichment analysis on the promoters of the light-genes. Previous studies show that CRE-mediated gene activation is one of the most important pathways for IEG expression [46], [47], [48] and for light-induced expression of *per1* and *per2* [49]. CRE's are present in the promoters of many light-induced genes [2]. Indeed, we found CRE-like sequences in 18 of the 30 light-induced genes. Using the definition of the promoter region used by PAINT, none of the light-induced clock genes (*per1*, *per2*, and *rora*) contained identifiable CRE sequences in their promoters (File S1). It is important to note, however, that both *per1* and *per2* contain functional CREs that lie further upstream in their promoters [49]. This reveals a limitation of the PAINT analysis, in that enhancer elements may reside >1000 bp from the transcriptional start site. This possibility is especially significant since chromatin conformation mapping reveals distant regulatory elements [50], [51]. Nevertheless, analysis of proximal promoter regions has a high probability of revealing functionally relevant elements, as the CRE family is significantly enriched in the light pulsed genes (Table S5). In cases where an expected TRE is not found (e.g., light-induced signaling genes *prkca*, *prkcb*, and *rasa2* that lack a CRE site), the possible TRE may lie outside the 1100 bp sequence used in PAINT. It is also possible that there may be alternate transcriptional start sites not included in the analysis (e.g., *per1*), or that alternative activation pathways may be used.

Besides CRE, PAINT analysis also revealed SRE, E2F, PAX3, and GABP binding sites as significantly enriched in the promoter of light-induced genes. While SRF and E2F sites are relatively rare in these promoters, GABP and PAX3 sites are widely present (Figure S4). These TREs appear to augment light-induced activation. While CRE alone is capable of driving light-induced activation, it is often not sufficient to do so (Figure 7). For example, *vip* and *grp* genes have highly conserved CRE sites near the transcriptional start sites, yet neither gene showed any response to light exposure. Conversely, we found that having GAPB and PAX3 strongly enhanced the light induced activation. Many well known immediate early genes (*fos*, *jun*, *junb*, *egr1*, *egr2*, and *dusp1*) have either one or both of these TREs in their promoter. *Per1* and *per2* would also fall into this category of genes if we considered the CREs that are located outside the promoter as defined for our PAINT analysis. This significant enrichment of multiple types of TREs suggests co-regulation of transcriptional activation. It is of interest to note that PAX3, GABP, and E2F have all been associated with cell differentiation. GABP in particular has been linked to CRE-regulated transcriptional regulation, through interaction with CREB binding protein (CBP/P300) [52], [53]. It is therefore possible that GABP has a direct role in CREB-mediated gene activation in SCN. Having these sites present in the promoter elements of light activated genes implies light response in the SCN is through a complex and highly coordinated network of gene regulation.

In additional to network analyses, we also investigated the expression profiles of individual genes. Our study limits itself to a manageable size by using two time points-ZT 6 and ZT 15 and exposure to light at night-with the expectancy of observing previously reported rhythmic profiles for several genes in the context of the larger system, and thus describe their systematic time- and region-specific regulation. We performed statistical tests through standard ANOVA with estimating FDR. However, as FDR correction is based on the assumption that gene expression measures are independent and correlated, we found that ANOVA with individual p-values provides a more stringent test. Recent developments indicate that this approach is fruitful even when scaled up to tens of thousands of genes measured using microarrays and in the extreme case consider all the data from expressed genes in clustering and pattern or module identification e.g., Weighted Gene Correlation Network Analysis (WGCNA): [54], [55], [56]. Hence, our approach biases towards reducing false negatives at the key first step of identifying differential gene expression profiles.

Our results show that the SCN has a highly dynamic and specific expression profiles. First, there are distinctions between the SCN and the surrounding hypothalamus tissue: 49 of the 89 genes we tested show enriched expression in the SCN (Figure S5). The most highly SCN-enriched genes are the neuropeptides *avp*, *vip*, and *grp*, and their receptors (*avpr1a*, *grpr*, *vipr2*). This confirms the local enrichment of these SCN neuropeptides. Clock genes *per2*, *cry1*, *rora*, *rorb*, and *rasd1* are also highly SCN-enriched, supporting the specific role of SCN as the master clock. Of the genes enriched in the SCN, many show time-dependent variation in expression levels as well as strong acute response to light at night. This is in contrast to the expression profiles in the surrounding hypothalamus, where little variation can be seen between the two time points we sampled, or between the night samples and night samples exposed to light. The two time points studied are unlikely to capture all the genes at their peak and trough of expression, and thus our design likely underestimates rhythmicity. Nevertheless, the overall lack of diurnal or light-induced variation in non-SCN hypothalamus reaffirms the specificity of the SCN.

As noted above, our study design likely underestimates the extent of rhythmically expressed genes. For example, the levels of *per2*, which is well-established as a rhythmic gene in SCN, did not differ between ZT 6 and ZT 15 in SCN shell samples in this study, while in the core, *per2* levels were higher at ZT 15 than at ZT 6. Thus, it is important to keep in mind that the phases selected for study may miss rhythmically expressed genes. Similarly, the use of a single time-point (1 hr after the start of the light pulse) in the ZT 15+LP group may underestimate the temporal cascades of gene expression that result.

Within the SCN, our results confirm that the neuropeptides *vip* and *grp* clearly mark the locations of the core, while *avp* marks the shell. Strikingly, the majority of the genes differentially expressed between the two regions are shell-enriched, even though the core region receives direct neuronal input from the retina, and is more affected by light. This is consistent with the proposed role of the shell as the output region of the SCN, and with higher amplitude gene expression rhythms [16]. The profile of differential gene expression we observed reaffirms the unique functionalities of SCN core and shell.

Besides the differences in gene expression between anatomic core-shell regions, our results also show striking SCN responses to a 1-hr light pulse at night. Light-induced expression occurred in four types of genes: transcription factors, signal transduction genes, genes that affect chromatin structure (*kdm6b* and *gadd45b*), and genes affecting mRNA splicing (*ccnl1*). The transcription factors, chromatin remodeling and splicing-related genes likely enable neurons to activate expression of secondary response genes. The induction of signal transduction genes, conversely, may serve to limit the molecular response to light. Several of the light induced genes negatively regulate the MAPK pathway. For example, *dusp1* and *dusp4* encode phosphatases that inactivate MAPK by dephosphorylation, and *rasa1* and *rasa2* products inhibit *ras* function. These inhibitory activities, occurring at different levels of the MAPK pathway, may contribute to the transient nature of most IEG inductions: gene activation apparently blocks signaling through the pathways that induced them. Combined with the inherent instability of IEG transcripts, this inhibition of signaling would limit the duration and extent of IEG induction. Interestingly, we have also observed a self-limiting response in the CREB/MAPK pathway through negative regulation. Of the genes showing lower levels of expression after light exposure at night, one of them is *creb1*, which decreased in expression in the shell. Since CREB is presumably the key to the light-induced response [47], such negative regulation might provide a feedback mechanism to limit the transcriptional response within the shell region following light exposure. Thus, light exposure simultaneously activates response genes, including immediate early genes and light-responsive clock genes, and also down-regulates the MAPK and CREB cascades responsible for their activation. Our multigenic analysis approach supports definition of this system behavior. Whether a strong but physiologically relevant stimulus leading to activation of gene expression cascades in other neuronal systems similarly leads to ‘molecular recurrent inhibition’ is a question for which the analysis of gene regulatory programs seems ideally suited.

In summary, our present work provides a unique approach toward elucidating the inner workings of the circadian clock within the SCN. Detailed tracking of light-induced events not only provides information on how SCN neurons respond to stimuli but also provides insight on core-shell communication. Although, our findings on SCN functional networks are potentially limited to the specific time points we have chosen to study, the success of this initial phase of our project serves as a proof of principle for data acquisition and analysis in small brain regions by combining analysis of a high-dimensional gene expression dataset with anatomical resolution and levels of sample replication typical of *in situ* hybridization. The SCN, a neural site for which a great deal is already known about temporal regulation and light inducibility, is an ideal candidate for building a network model of neuronal transcriptional interactions.

## Materials and Methods

*This study was performed in strict accordance with the recommendations in the Guide for the Care and Use of Laboratory Animals of the National Institutes of Health. The protocol was approved by the Institutional Animal Care and Use Committee (IACUC) of Thomas Jefferson University.*

### Animal preparation

4–6 week old male C57BL/6J mice were purchased from Charles River (Wilmington, Massachusetts). The animals were housed with 12-hour light, 12-hour dark cycles. During the light phase of the lighting cycle, light (150 lux) was provided by warm white fluorescent bulbs. Animals were entrained for 10 days with free access to food and water. On the day of experiment, animals were given a one-hour light exposure (150 lux of white light) at ZT 14, 2 hours into their regular dark period, and sacrificed one hour later at ZT 15. SCNs were also collected from non-light-pulsed animals at ZT 15, and from animals during the light period (ZT 6). Animals were euthanized by CO_2_ asphyxiation in prevailing lighting condition (dim red light for ZT 15). Their brains were extracted, and the hypothalamic blocks were dissected. Blocks were embedded in OCT compound, and frozen on dry ice. Animal experiments were carried out at Thomas Jefferson University and were approved by the Institutional Animal Care and Use Committee (IACUC).

### Staining and Histology Analysis

The staining protocol was adapted from a standard hemotoxylin/eosin stain to minimize RNA degradation and to enable laser capture. The embedded hypothalamus blocks were sectioned in a cryostat at 12 µm thickness, and thaw mounted on glass slides. The sections were first fixed in 75% ethanol for 30 sec, then briefly rinsed in water and stained in hemotoxylin (Sigma-Aldrich, St. Louis, MO) for 30 sec. Next, the slides were briefly rinsed again in water mixed with Scott's tap water substitute (Electron Microscopy Sciences, Hatfield, PA) for color development, and counterstained in eosin/ethanol mixture (1∶1 v/v) (Sigma-Aldrich) for 20 sec. The slides were then put through a standard dehydration process (50% ethanol, 30 sec; 75% ethanol, 30 sec; 95% ethanol 30 sec; and 2×100% ethanol, 30 sec each). The slides were then rinsed briefly in xylenes (Sigma-Aldrich) and transferred into fresh xylenes for 3 min to further remove trace of ethanol. Finally, the slides were air dried for 5 min for immediate LCM capture.

### LCM and Sample Preparation

LCM was performed with the Arcturus PixCell II (Life Technologies, Carlsbad, CA). The location of the SCN was determined by enriched nuclei staining and physical location (Figure 2, top panel). The core and shell of the SCN were collected from non-overlapping ventral-lateral and dorsal-medial regions, respectively (Figure 2), based on a conservative estimation of existing literature [16], [17], [18], [32], [33]. Control hypothalamus samples were collected from the same section at lateral regions immediate outside of the SCN (not shown). The samples were collected on CapSure Macro LCM caps (Life Technologies). Regional samples from five tissue sections per SCN were collected and pooled. Approximately 500 cells were collected for each sample. RNA was extracted using the PicoPure RNA isolation kit (Life Technologies) following the manufacturer's instructions, incorporating the optional DNase treatment. The RNA samples' quantity and quality were checked using the Bioanalyzer (Agilent, Santa Clara, CA). Average yield was about 3 ng of RNA per sample.

### cDNA Preparation and High Throughput Quantitative Real-time PCR

A standard reverse transcription protocol was used to generate cDNA from RNA samples. Random primers were used. cDNA was then pre-amplified with a pool of 96 pairs of PCR primers using TaqMan PreAmp Master Mix (Applied Biosystems, Foster City, CA), as required by the BioMark high-throughput qPCR protocol (Fluidigm, South San Francisco, CA). The following thermo cycles were used: 95°C for 10 min, 16 cycles of 95°C for 15 sec and 60°C for 2 min.

High-throughput quantitative PCR on pre-amplified cDNAs was performed using BioMark system (Fluidigm) according to the manufacturer's instructions. The same primer set used in pre-amplification was used for probe-based quantitative PCR. 96.96 dynamic arrays for gene expression were used. qPCR is based on the Universal Probe Library (Roche, Indianapolis, IN). Detailed PCR primer and probe information is listed in Table S1. These gene/primer pairs were pre-validated by both standard PCR, and qPCR analysis using cDNA generated from mouse hypothalamic RNA (Clontech, Mountain View, CA).

### Data analysis

High-throughput qPCR output was analyzed using the BioMark system software. Cycle threshold (C_T_) for each gene assay was determined by the software automatically, using linear derivative baseline and a minimum quality score of 0.65. Of the 96 genes pre-amped, two (*bhlhe41* and *avpr1b*) failed to produce consistent results and were removed from further analysis. Expression data for housekeeping genes *atp5p* and *hprt* were also excluded due to several failed reactions that preventing their use as normalization controls. Each PCR reaction was also manually inspected for abnormal amplification profiles; the C_T_ values were then exported for data analysis. The average C_T_ numbers of the remaining 3 housekeeping genes (*gapdh*, *tbp*, and *actb*) were used for normalization. −ΔC_T_ was calculated for each assay. Statistical comparisons were done using the −ΔC_T_ values. Complete results are listed in Table S7.

Genes showing significant changes between time points, light treatment, and regional variations were identified using ANOVA. Pairwise comparisons of core vs. shell, ZT 15 vs. ZT 15+LP, day (ZT 6) vs. night (ZT 15) were performed using post-hoc *t*-test. Multiple testing corrections based on estimated false discovery rate (FDA) was performed using Partek data analysis software (Partek, St Louis, Missouri) as implemented in the q-value. Using this approach with conventional FDA thresholds of 10–20%, 60–80% of the genes show a statistically significant time effect (including light treatment) and 40–50% of the genes show a statistically significant regional effect (core vs. shell). Both are higher than the results obtained through ANOVA (Table S2). Therefore, ANOVA with p-values less than 0.05 was a more stringent inclusion criterion than ANOVA with FDR correction, and was used in all subsequence analysis.

The hierarchical clustering and principal component analyses were done in MeV [57], [58] with Pearson Correlation.

### Functional annotation using DAVID and Cytoscape

A baseline functional network map was constructed for the genes included in our qPCR analysis. The baseline network was constructed using the DAVID Bioinformatics Resource v6.7 to identify the relevant annotation terms, and the genes from the tested assays assigned to each. The network map is a visual representation, using Cytoscape software [59], [60], of those annotation terms and gene assignments. In the network, each annotation term is represented as an individual node (square). For each term, the size of its node in the network is proportional to the number of tested genes assigned to the group. Connections are drawn between annotation terms whenever one tested gene is assigned to both annotation terms. The connections between nodes have scaled thickness that increases with the number of shared genes between the two annotation terms. In this manner it is possible to view the tested assays not as a gene list, but rather as a network of related gene products and cellular functions.

Cytoscape was further used to plot subsets of genes identified by our comparison analyses upon the base representation of the entire network. For each gene subset of interest, only the statistically identified, differentially expressed genes were included. Each gene with either a significant treatment effect, or a significant treatment by region interaction, was identified based on its ΔΔC_T_ values. The genes with significant alteration for each treatment were then superimposed on the baseline function map. Nodes and lines not represented within the gene subset were removed from the network. This format allows for dynamic visualization of the differentially expressed genes in terms of the functional effects of their products. Gene subsets that were selected for representation in Cytospace maps were those genes enriched in shell (relative to core); elevated at ZT 6 (vs. ZT 15); elevated at ZT 15 (vs. ZT 6); and light-induced genes (in either core or shell region, ZT 15+LP>ZT 15).

### Analysis of Transcriptional Regulatory Element (TRE) Frequency

Promoter Analysis and Interaction Network Toolkit (PAINT, v.4.0; [37], [38]) was used for analysis of Transcriptional Regulatory Element (TRE) frequency. The location of the transcription start site was defined using mouse Reference Sequences Collection [61]. Promoter sequences were manually retrieved from genome assembly using the range of −1000 bp to 100 bp from the transcriptional start site. The location of the promoter for each gene analyzed by qPCR is listed in Table S8 and the corresponding FASTA sequence is listed in File S2. Whole-genome estimates of TRE frequencies in all promoters were generated by PAINT. Default settings were used for TRE identification based on TRANSFAC database. Statistical comparison of TRE frequency in the subset to TRE frequency in reference sets (the whole genome or to the set of 89 investigated genes) was performed using Fisher's Exact Test (FET). Network of enriched TREs and related genes was visualized using Cytoscape. TREs were grouped based on the corresponding transcription factors.

## Supporting Information

Figure S1 **Regional specific expression of SCN neuropetides.** Distinct levels of expression of the SCN neuropeptides vip, grp, and avp were shown in the core and the shell, indicating the specificity of the laser capture techniques. The numbers shown here were the normalized HTqPCR results (Calculated −ΔC_T_ using the average C_T_ of *gapdh*, *tbp*, and *actb* of each sample). Error bars indicate the standard errors of the mean. Direct comparison of these expression levels were also shown in Figure 5B.
(TIF)

Figure S2 **Normalization comparison using different control genes.** HTqPCR results were normalized by calculating −ΔC_T_ using the average C_T_ of *gapdh*, *tbp*, and *actb*, or each of the three genes individually. Results are hierarchically clustered. The sample trees for each normalization are shown. Square indicates SCN core samples; triangle, SCN shell; circle, hypothalamus regions outside SCN. Green indicates samples collected at ZT 6; blue, samples collected at ZT 15; red, light pulsed samples collected at ZT 15. Very similar results, especially for the SCN samples, were obtained.
(TIF)

Figure S3 **Differential Expression in SCN (complete result).** Expression differences (−ΔΔC_T_) are shown. A) The presence or absence of a light pulse at ZT 15 significantly affected the expression of 32 genes in the SCN. The genes are ordered based on the −ΔΔC_T_ values. All genes are shown in ranking of expression differences. B) A total of 29 genes showed significant differential expression between core and shell of the SCN. C) A total of 26 genes showed significant differences between day (ZT 6) and night (ZT 15). Black columns indicate significant differences (ANOVA with post hoc *t*-test, p<0.05) Error bars indicate standard errors.
(TIF)

Figure S4 **Significantly enriched TREs.** TF binding site enrichment analysis on light-induced genes was performed using PAINT v4.0. The promoter of the 89 genes was used as background. Significantly enriched TREs are shown. Black boxes indicate the presence of individual TRE. Complete statistical results are provided in.
(TIF)

Figure S5 **Gene expression clustering.** The normalized qPCR results (in −ΔC_T_) were median centered and hierarchical clustered for the genes. Red represents elevated expression and green represents lower expression. Genes are loosely clustered into three groups based relative expressions between SCN and the surrounding hypothalamus (SCN enriched, hypothalamus enriched, and evenly expressed). Genes that respond strongly to light exposure at night (ZT 15+LP) can be visualized in the SCN core samples.
(TIF)

Table S1 **Genes used in Biomark analysis.** List of genes used the BioMark. Information include: office gene symbol, gene name, gene_ID, accession numbers, gene functions inferred from literature search, forward/reverse primers for qPCR, UPL used, and relevant literature.
(XLS)

Table S2 **ANOVA results with multiple testing and FDR.** Results were generated using PARTEK software.
(XLS)

Table S3 **DAVID annotation of the 89 genes analyzed.** Gene functions based on terms retrieved through DAVID.
(XLS)

Table S4 **List of identified TREs and results of enrichment analysis.** TRE names and consensus sequences are listed. Three sets of Fisher Exact Test results are shown. 1) TREs in all 89 analyzed genes, using entire mouse genome as background. 2) TREs in the light-induced genes, using entire mouse genome as background. 3) TREs in the light-induced gene set, using the promoters of the 89 genes as background. Significant scores (p<0.05) are highlighted in bold. Empty spot indicate a particular TRE is not present in the light-induced genes.
(XLS)

Table S5 **TRE family enrichment analysis of light-induced genes.** TRE families with same transcription factor were grouped. FET test was performed using the 89 genes as background.
(DOC)

Table S6 **Lists of significantly regulated genes for DAVID mapping.** Note: Although CRE family of TREs is significantly enriched, each CRE by itself is not. Therefore, none of the CRE site is shown here.
(XLS)

Table S7 **Normalized qPCR data and Standard error analysis.** Raw data from BioMark were normalized (−ΔΔC_T_). Average and SEM of each animal group are also shown.
(XLS)

Table S8 **Location of the Promoter Sequences used for PAINT.** Location of the promoters were defining using transcription start site of the RefSeq for each gene. The location information (listed in File S1) was then used to retrieve corresponding genomic sequences for PAINT analysis.
(XLS)

File S1 **Identified TREs.** TREs were identified using the MATCH software which is built in the PAINT tool with the Transfac database. The names of the TREs and corresponding transcription factors are listed. Sequences are arranged so position 1000 is the transcription start site. Position 0 is 1000 bp upstream and position 1100 is 100 bp of the transcript. +refer to direction of the transcription-refer to the direction opposite of the transcription.
(TXT)

File S2 **Promoter Sequences.** Promoters defined as the regions between 1000 bp upstream of transcription start site to 100 bp of the transcript. The transcription start sites were determined using RefSeq sequences. The sequences are in FASTA format.
(TXT)
