# Supplementary figures and images for: Integrative Gene Regulatory Network Analysis Reveals Light-Induced Regional Gene Expression Phase Shift Programs in the Mouse Suprachiasmatic Nucleus
Source: PLoS One. 2012 May 25;7(5):e37833. doi: 10.1371/journal.pone.0037833 (PMC3360606; doi:10.1371/journal.pone.0037833)

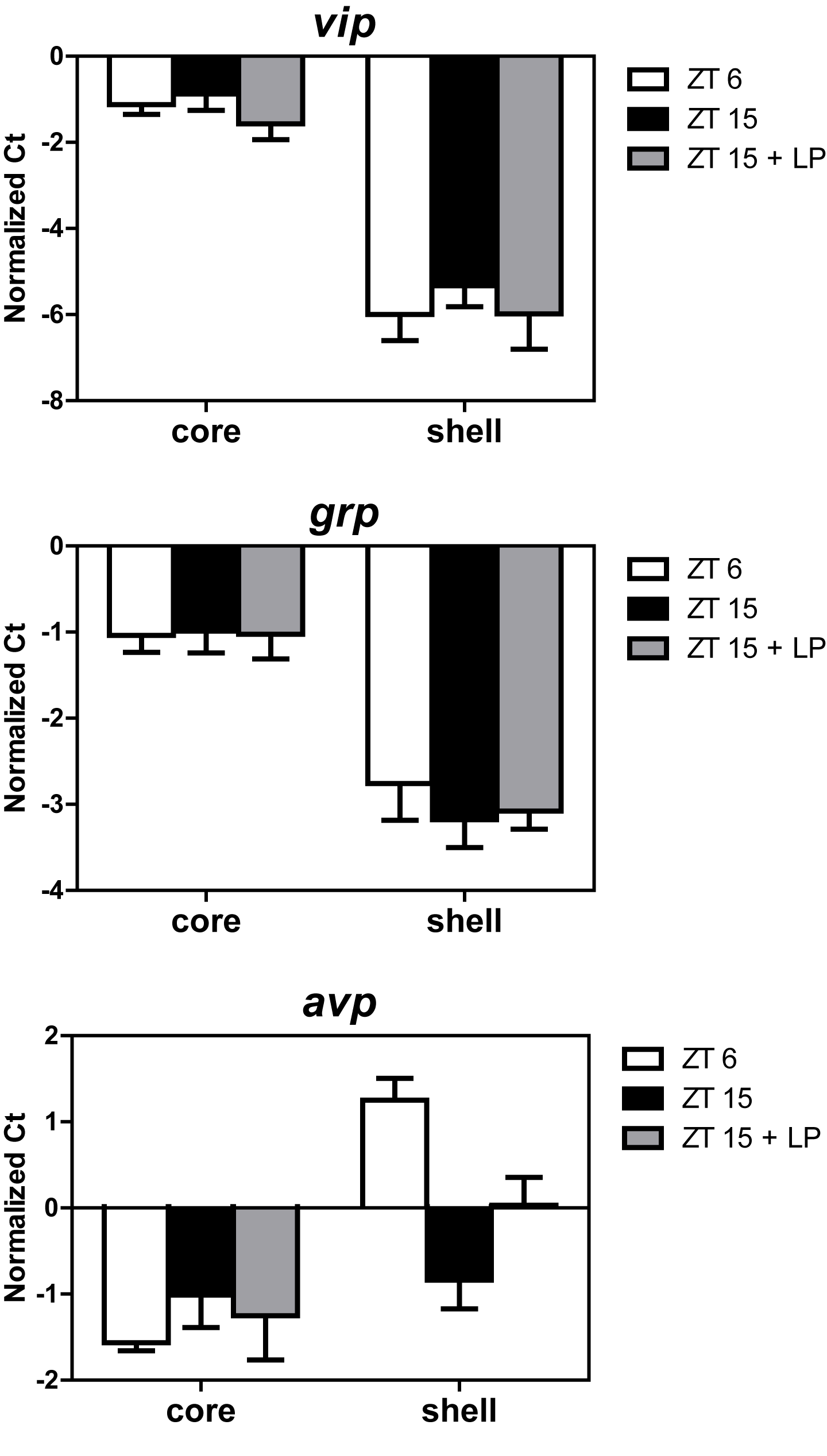

Supplement: Figure S1 — Regional specific expression of SCN neuropetides. Distinct levels of expression of the SCN neuropeptides vip, grp, and avp were shown in the core and the shell, indicating the specificity of the laser capture techniques. The numbers shown here were the normalized HTqPCR results (Calculated −ΔCT using the average CT of gapdh, tbp, and actb of each sample). Error bars indicate the standard errors of the mean. Direct comparison of these expression levels were also shown in Figure 5B. (TIF) [file pone.0037833.s001.tif]

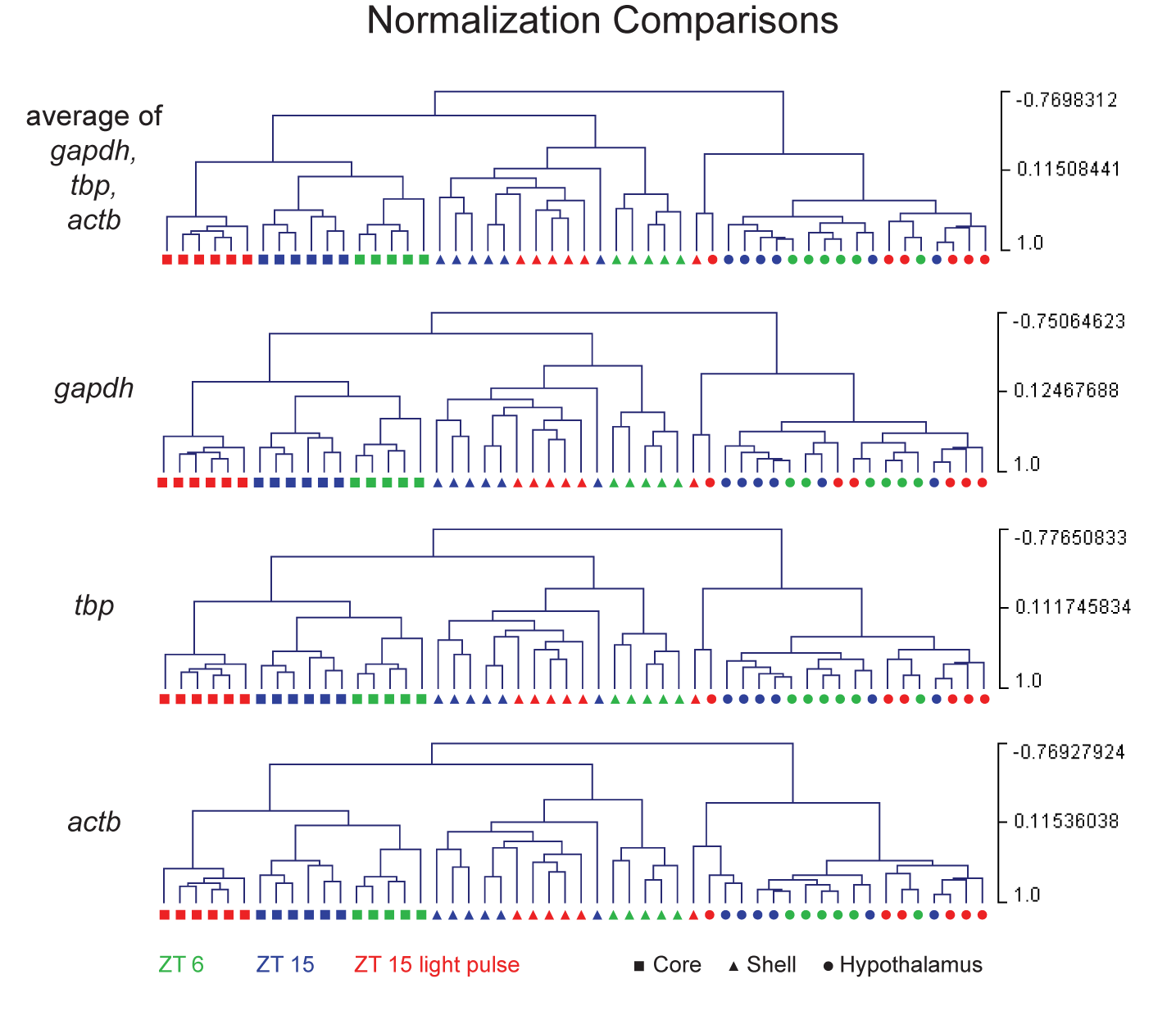

Supplement: Figure S2 — Normalization comparison using different control genes. HTqPCR results were normalized by calculating −ΔCT using the average CT of gapdh, tbp, and actb, or each of the three genes individually. Results are hierarchically clustered. The sample trees for each normalization are shown. Square indicates SCN core samples; triangle, SCN shell; circle, hypothalamus regions outside SCN. Green indicates samples collected at ZT 6; blue, samples collected at ZT 15; red, light pulsed samples collected at ZT 15. Very similar results, especially for the SCN samples, were obtained. (TIF) [file pone.0037833.s002.tif]

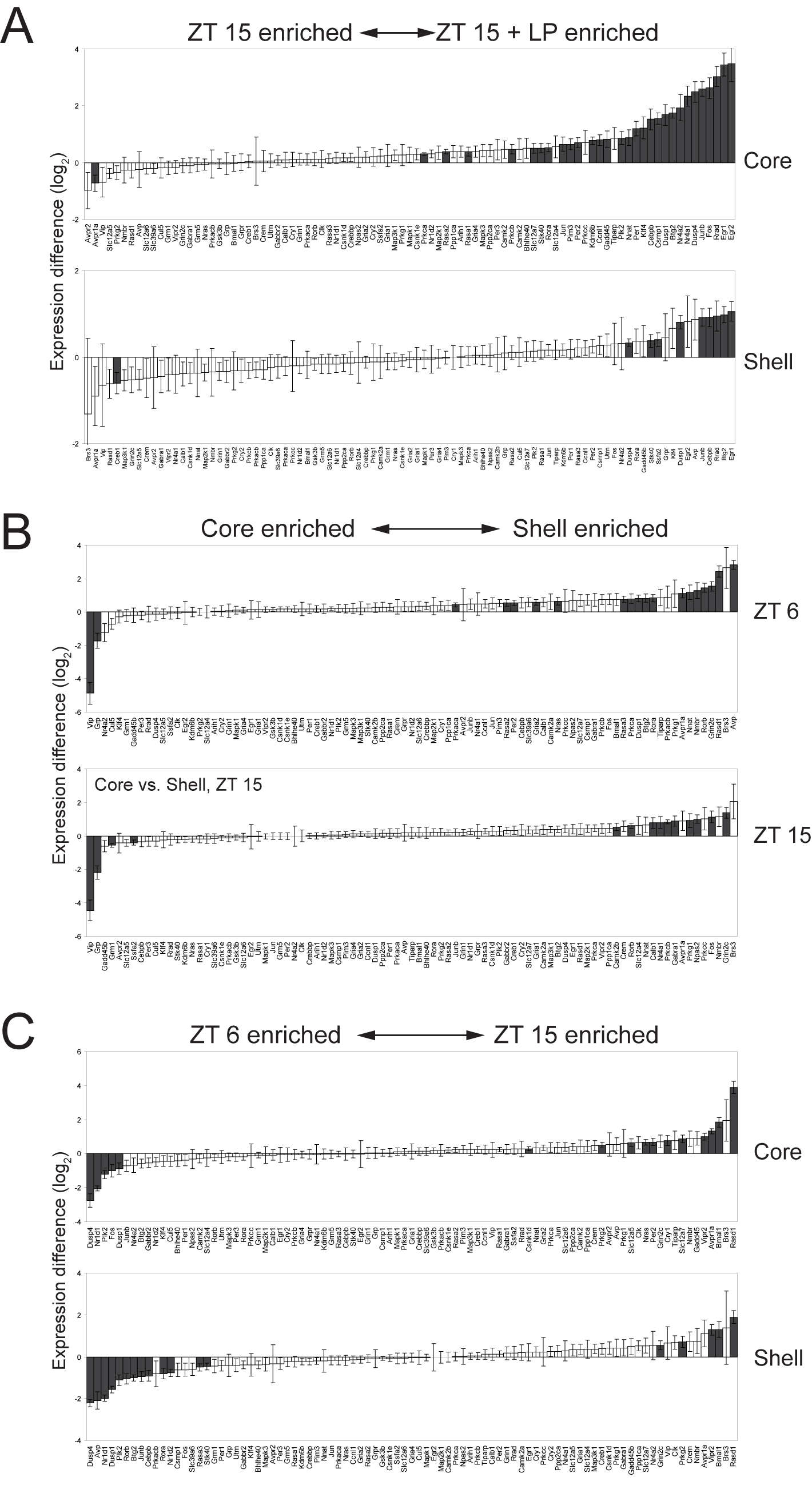

Supplement: Figure S3 — Differential Expression in SCN (complete result). Expression differences (−ΔΔCT) are shown. A) The presence or absence of a light pulse at ZT 15 significantly affected the expression of 32 genes in the SCN. The genes are ordered based on the −ΔΔCT values. All genes are shown in ranking of expression differences. B) A total of 29 genes showed significant differential expression between core and shell of the SCN. C) A total of 26 genes showed significant differences between day (ZT 6) and night (ZT 15). Black columns indicate significant differences (ANOVA with post hoc t-test, p<0.05) Error bars indicate standard errors. (TIF) [file pone.0037833.s003.tif]

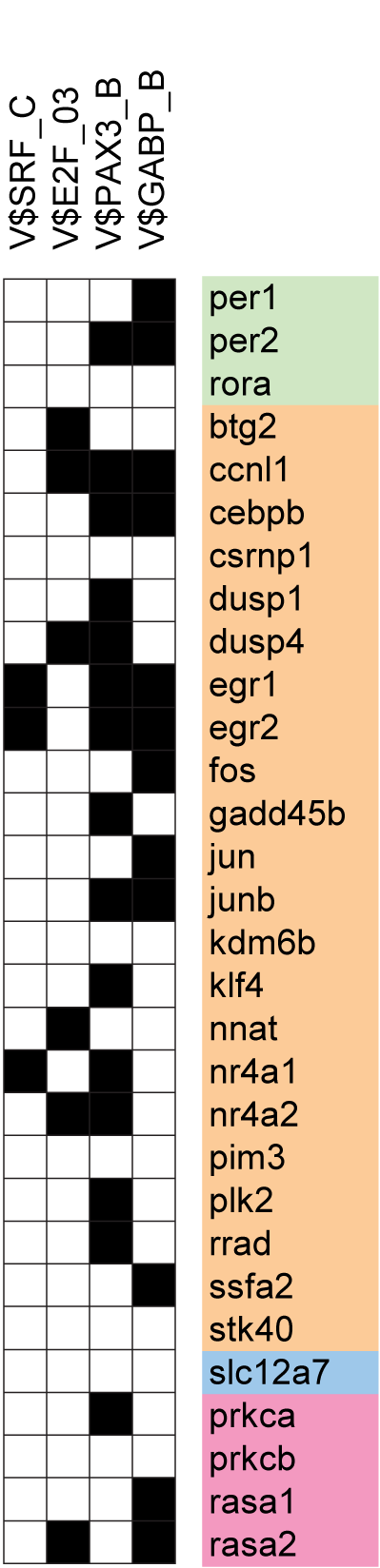

Supplement: Figure S4 — Significantly enriched TREs. TF binding site enrichment analysis on light-induced genes was performed using PAINT v4.0. The promoter of the 89 genes was used as background. Significantly enriched TREs are shown. Black boxes indicate the presence of individual TRE. Complete statistical results are provided in. (TIF) [file pone.0037833.s004.tif]

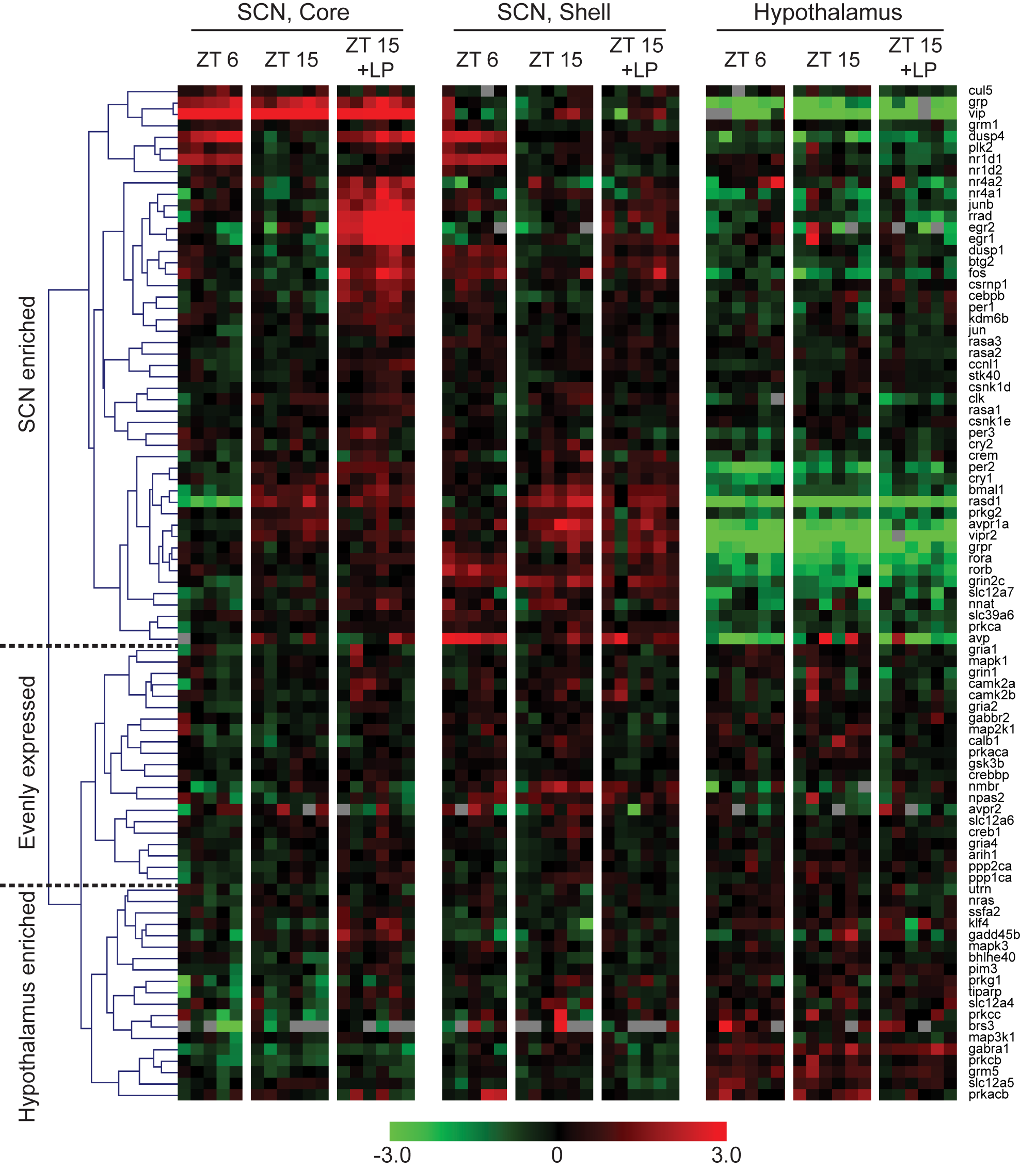

Supplement: Figure S5 — Gene expression clustering. The normalized qPCR results (in −ΔCT) were median centered and hierarchical clustered for the genes. Red represents elevated expression and green represents lower expression. Genes are loosely clustered into three groups based relative expressions between SCN and the surrounding hypothalamus (SCN enriched, hypothalamus enriched, and evenly expressed). Genes that respond strongly to light exposure at night (ZT 15+LP) can be visualized in the SCN core samples. (TIF) [file pone.0037833.s005.tif]
