# Supplementary material for: Integrative Gene Regulatory Network Analysis Reveals Light-Induced Regional Gene Expression Phase Shift Programs in the Mouse Suprachiasmatic Nucleus
Source: PLoS One. 2012 May 25;7(5):e37833. doi: 10.1371/journal.pone.0037833 (PMC3360606; doi:10.1371/journal.pone.0037833)
Supplement: Table S5 — TRE family enrichment analysis of light-induced genes. TRE families with same transcription factor were grouped. FET test was performed using the 89 genes as background. (DOC) [file pone.0037833.s010.doc]

**Supplemental Table 8. TRE family enrichment analysis of light-induced genes**

| **TF** | **TRE families** | **FET p-value*** |
| --- | --- | --- |
| CREB | CRE | **0.007** |
| SRF | SRE | 0.109 |
| AP-1 | AP-1 | 0.664 |
| EGR | EGR | 0.730 |
| E-box | E-Box | 0.547 |
| E2F | V$E2F_03 | **0.036** |
| PAX3 | V$PAX3_B | **0.042** |
| GABP | V$GABP_B | **0.011** |

* Using TREs from the 89 genes tested as reference set
